# Inclusion of Ethanol Extract of Mesquite Leaves to Enhance the Oxidative Stability of Pork Patties

**DOI:** 10.3390/foods8120631

**Published:** 2019-12-02

**Authors:** Margarita Irene Ramírez-Rojo, Rey David Vargas-Sánchez, Brisa del Mar Torres-Martínez, Gastón Ramón Torrescano-Urrutia, José Manuel Lorenzo, Armida Sánchez-Escalante

**Affiliations:** 1 Coordinación de Tecnología de Alimentos de Origen Animal (CTAOA), Centro de Investigación en Alimentación y Desarrollo, A.C. (CIAD), Carretera Gustavo Enrique Astiazarán Rosas, 46, Hermosillo, Sonora 83304, Mexico; margaritaramirezrojo@gmail.com (M.I.R.-R.); rey.vargas@ciad.mx (R.D.V.-S.); brisa.torres@estudiantes.ciad.mx (B.d.M.T.-M.); gtorrescano@ciad.mx (G.R.T.-U.); 2 Centro Tecnológico de la Carne de Galicia, Rúa Galicia N° 4, Parque Tecnológico de Galicia, San Cibrao das Viñas, 32900 Ourense, Spain

**Keywords:** sensorial properties, colour parameters, conjugated dienes, polyphenol content

## Abstract

The lipid oxidation (LOX) of pork meat has been associated with loss of quality and shorter shelf life. Consequently, synthetic antioxidants have been used to reduce this process, but their use has shown potential health risks. Thus, the use of natural ingredients has been suggested as a strategy to prevent LOX. This study aimed to assess the oxidative stability of pork patties treated with ethanol extract of mesquite leaf (EEML) during storage. Furthermore, the polyphenol composition (TPC, total phenolic, TFC, total flavonoid) and antioxidant activity (antiradical and reducing power activity) of EEML were also evaluated. For this study, five treatments (CN (control), without antioxidant; Asc, ascorbic acid 0.02%; BHT, butylated hydroxytoluene 0.02%; EEML1, 0.05%; and EEML2, 0.1%) of pork patties were applied. Patty samples were stored at 4 °C, and physicochemical parameters, lipid oxidation, total antioxidant capacity of the meat, and sensory analysis were evaluated at 0, 3, 7, and 10 days of storage. EEML presented high values of TPC (278.5 mg gallic acid equivalent (GAE)/g) and TFC (226.8 mg rutin equivalents (RE)/g) levels. The addition of EEML did not modify the chemical composition of the pork patties. On the other hand, colour parameters were affected by the inclusion of EEML in pork patties, presenting the lowest a* in the CN group compared to the other groups after 10 days storage. Lipid oxidation increased during the whole period, showing the lowest (*P* < 0.05) conjugated dienes and thiobarbituric acid reactive substances (TBARS) values (40% and 90% of inhibition, respectively) compared to the CN group. Regarding sensory analysis, there were no significant differences in colour, appearance, odour, flavour, juiciness, fat sensation, and firmness of the cooked pork patties among treatments. These results suggest that EEML has great potential as a natural antioxidant for meat products.

## 1. Introduction

In Mexico, pig production is one of the most important activities in the livestock sector, and recently pork became the second most popular meat in this country, with a per capita consumption of 11.6 kg in 2017, with a total domestic consumption of 2.4 M metric tonnes [1]. Moreover, pork meat plays an important role in human nutrition as a source of amino acids, minerals, vitamins, and fatty acids [2,3]. Fatty acids (FA) are an important source of energy for human consumption. However, it is recommended to increase the dietary intake of monounsaturated (MUFA) and polyunsaturated (PUFA) fatty acids and to reduce saturated fatty acids (SFA) [4,5]. Nevertheless, the high PUFA content in pork meat and meat products results in low oxidative stability [6,7,8].

Lipid oxidation is one of the most important processes occurring in the food matrix responsible for the deterioration of the quality and thus shortening the shelf life [9]. In this regard, the lipid oxidation process (LOX) is responsible for off-flavours and unacceptable taste as well as discolouration, loss of nutritional value, the formation of toxic compounds, drip loss, etc., which could affect the acceptance of the product by the consumer [10,11,12]. Thus, synthetic antioxidants such as butylated hydroxyanisole (BHA) and butylated hydroxytoluene (BHT) have been used to reduce LOX. However, their use has decreased due to adverse effects on consumer health [13,14]. The addition level is recommended to be no more than 200 ppm on the fat level and 30 ppm on the meat weight basis. Due to the potential toxicological effects of synthetic antioxidants, natural antioxidants are an interesting alternative to conventional antioxidants [15,16,17,18]. In order to reduce LOX, the use of natural ingredients such as phenolic compound extracts from plants (seeds, peels, barks, woods, flowers, and leaves) has been proposed [19,20,21,22]. Mesquite (*Prosopis* spp.) has long been a useful biotic resource for the people of the arid and semiarid regions in Mexico. The mesquite wood, pods, and leaves are commonly used in human and animal food and medicinal treatment [23]. Moreover, it has been reported that extracts obtained from the bark, pods, pollen, and leaves of plants from the genus *Prosopis* have bioactive properties. Their antifungal, antimicrobial, anti-tumoral, anti-inflammatory, antihyperlipidemic, and antioxidant properties are attributed to their phytochemical content [24]. However, the use of mesquite as a natural source of antioxidant compounds for meat products and its potential health benefits are not well studied. Therefore, the aim of this study was to evaluate the effect of ethanol extract of mesquite leaf (EEML) on the oxidative stability of pork patties during storage.

## 2. Materials and Methods

### 2.1. Chemicals and Reagents

All the chemical products used were of analytical grade. Folin–Ciocalteu reagent, sodium carbonate, aluminium chloride, 1,1-diphenyl-2-picrylhydrazyl (DPPH^•^), ethanol, hexane, 2-propanol, methanol, sodium phosphate, potassium ferricyanide, iron chloride, gallic acid, quercetin, butylated hydroxytoluene, and ascorbic acid were purchased from Sigma Chemicals (St. Louis, MO, USA). Whereas, 2-thiobarbituric acid and trichloroacetic acid were obtained from J.T. Baker ^®^.

### 2.2. Extract Preparation

Mesquite leaves (*Prosopis velutina*) were collected in the Northwest of Mexico (Sonora state, Ures municipality; 29°7’19.72′N, 110°16’58.35′ W; 476 m a.s.l.) and botanically identified by a specialist from the Sonora University Herbarium (plant number 26120). Mesquite leaves were washed with distilled water, dried at room temperature (25–30 °C) and milled (20 mesh) for subsequent phenolic extraction [25]. Mesquite leaf powder was extracted with ethanol (1:10) by ultrasound-assisted extraction (42 kHz/25 °C/30 min). Subsequently, the mixture was centrifuged (4200× *g*/10 min) to obtain the supernatant, followed by a second extraction process. Both solutions were filtered (Whatman 4 filter paper), concentrated under reduced pressure (Rotary evaporator BÜCHI R-200, Flawil, Switzerland), lyophilized (Freeze dryer Yamato DC401, Tokyo, Japan), and stored at −20 °C in the dark, until analysis.

### 2.3. Polyphenol Content

The total phenolic content (TPC) was determined by the Folin–Ciocalteu reagent method [26]. An aliquot of EEML (10 µL, 5 mg/mL) was homogenized with 160 µL of distilled water, 40 µL of Folin–Ciocalteu reagent (0.25 N), 60 µL of Na_2_CO_3_ (7%, *w*/*v*) and 80 µL of distilled water. After incubation (25 °C/in the dark/1 h), absorbance was measured at 750 nm in a spectrophotometer (Multiskan FC UV-Vis, Thermo Scientific, Vantaa, Finland) and the results were expressed as mg of gallic acid equivalent/g (mg GAE/g). The total flavonoid content (TFC) was based on the complex formation with aluminium chloride method [27]. EEML (10 µL, 5 mg/mL) was homogenized with 130 µL of methanol and 10 µL AlCl_3_ (5%, *w*/*v*). After incubation (25 °C/in the dark/30 min), the absorbance was measured at 412 nm and the results were expressed as mg of quercetin equivalent/g (mg QE/g).

### 2.4. Antioxidant Activity 

The antiradical activity was evaluated by the DPPH^•^ method [28]. EEML (100 µL, at 25, 50, and 100 µg/mL) were mixed with DPPH^•^ solution (300 μmol). After incubation (25 °C/in the dark/20 min), absorbance was measured at 517 nm, and the results expressed as a percentage of radical inhibition. The reducing power assay (RP) was measured by the Prussian blue method [29]. EEML (100 μL, 5 mg/mL) was mixed with 300 μL of phosphate buffer (0.2 M, pH 6.6), 300 μL of potassium ferricyanide (1%, *w*/*v*) and incubated in a water bath for 20 min (50 °C). Then, an aliquot of 300 μL of trichloroacetic acid (10%, *w*/*v*) was added and centrifuged (4200× *g*/10 min). Then, the supernatant was mixed with 100 μL of distilled water, and 250 μL of ferric chloride (0.1%, *w*/*v*), and the absorbance was measured at 700 nm.

### 2.5. Pork Patties’ Preparation and Storage

Minced pork meat (Semimembranosus m., 48 h post-mortem) was purchased from a local processor and mixed with salt (1.5%, *w*/*w*) and fat (10% in the final formulation, *w*/*w*). The mass was divided into five different treatments: (1) CN, control (without antioxidant); (2) Asc, ascorbic acid (0.02%, *w*/*w*); (3) BHT, butylated hydroxytoluene (0.02%, fat basis); (4) EEML1, ethanol extract of mesquite leaf (0.05%, *w*/*w*); and (5) EEML2, ethanol extract of mesquite leaf (0.1%, *w*/*w*). For each batch, a total of 14 patties (90 g) per treatment were prepared and placed on a Styrofoam tray. The trays with pork patties were wrapped with polyvinyl chloride film (17,400 cm^3^ O_2_/m^2^/23 °C/24 h). The patties were stored at 4 °C, in the dark for 10 days. At day 0, six patties per treatment were subjected to proximate analysis; while, eight patties were subjected to meat quality measurements during storage. At each sampling point (0, 3, 7, and 10 days), two packs of each treatment were assessed. 

### 2.6. Meat Quality Measurements

#### 2.6.1. Proximate Analysis 

Moisture, fat, ash, and protein content were determined following the standard procedures [30].

#### 2.6.2. Measurement of pH

The pH of the raw pork patties was measured after homogenization with distilled water at a ratio of 1:10, with a potentiometer (Model pH211, Hanna Instruments Inc., Woonsocket, RI, USA) with automatic temperature control [30].

#### 2.6.3. Colour Measurement

The colour measurement was performed using a spectrophotometer (model CM 508d, Konica Minolta Inc., Tokyo, Japan). The registered values were lightness (L*), redness (a*), yellowness (b*), Chroma (C*), and hue angle (h*). The pork patties were extracted from their packaging and exposed to atmospheric O_2_ for 30 min for blooming. Ten measurements were performed on the surface of each patty [31].

#### 2.6.4. Metmyoglobin Formation

The metmyoglobin formation (MMb) was estimated spectrophotometrically by measuring the reflectance at 525 and 572 nm with a spectrophotometer (model CM 508d, Konica Minolta Inc., Tokyo, Japan). The maximum value of the quotient K/S525 and K/S572 on the initial day of sampling (day 0) was fixed as 0% MMb, while 100% MMb was obtained after oxidizing the patties with a potassium ferricyanide solution (1%, *w*/*v*). Each value was the mean of 10 measurements on each pork patty’s surface [32].

#### 2.6.5. Water Holding Capacity

The water holding capacity (WHC) of the patties was determined gravimetrically [33]. Samples (10 g) were placed on fine mesh nylon, inserted into 50 mL tubes with a screwcap, and then centrifuged (4200× *g*/4 °C/10 min). The WHC was calculated using the following formula:

(1) $$\mathrm{WHC}\;(\%)\;=\;\frac{\mathrm{initial}\;\mathrm{weight}\;-\mathrm{weight}\;\mathrm{after}\;\mathrm{centifugation}}{\mathrm{initial}\;\mathrm{weight}}\;\times \;100$$

#### 2.6.6. Lipid Oxidation

The LOX was evaluated by the conjugated diene formation (CnD) [34]. Pork patties (0.5 g) were homogenized with 5 mL of hexane:isopropanol solution (3:2) for 1 min and centrifuged at 2000× *g*/4 °C/5 min. The absorbance was measured at 233 nm. Deionized water was used as blank, and CnD was quantified using a molar extinction coefficient of 25,200 M^−1^ cm^−1^. The results were expressed as µmol of CnD/mg of meat. The LOX was also measured by the thiobarbituric acid reactive substances (TBARS) formation [35]. Meat samples (10 g) were homogenized with 20 mL of trichloroacetic acid (10%, *w*/*v*) and centrifuged (2300× *g*/4 °C/20 min). Then, 2 mL of the filtered supernatant (Whatman 4 filter paper) was mixed with 2 mL of 2-thiobarbituric acid (20 mM) and boiled in a water bath for 20 min. After cooling, the absorbance was measured at 531 nm (Spectrophotometer Model 336001, Spectronic Genesys 5, Thermo Electron Corp., NY, USA). The malondialdehyde concentration (MDA) was calculated using a calibration curve, and the results expressed as mg of MDA/kg sample. 

#### 2.6.7. Total Antioxidant Activity of Meat Extract

The meat extract was obtained from 0.5 g of pork patties, which were homogenized with 5 mL of distilled water and centrifuged (4200× *g*/4 °C/10 min) [36]. The supernatant was used to determine the total antioxidant of the meat extract, measured by the DPPH• inhibition [28] and RP [29].

### 2.7. Sensory Evaluation

A sensory panel (*n* = 25, laboratory co-workers and students) was used to evaluate the sensory analysis of raw and cooked meat samples. Previously, uncooked pork patties were subjected to sensory evaluation of colour and appearance. Then, pork patties were grilled until they reached an internal temperature of 71 °C and subjected to sensory evaluation of colour, appearance, odour, flavour, juiciness, fat sensation, and texture. A descriptive seven-point scale was used (1 = extremely poor to 7 = excellent). 

### 2.8. Statistical Analysis

Three independent experimental trials (replications) were conducted, and the results presented as the mean ± standard deviation. Data of experimental patties were submitted to analysis of variance (ANOVA) according to a two factorial design using National Center for Social Statistics statistical software (NCSS, 2007) The treatments (CN, Asc, BHT, EEML1, and EEML2) and storage time (0, 3, 7, and 10 days) were the fixed terms in the model. For sensory evaluation, the panellists were considered a random factor as well. A Tukey–Kramer multiple comparison test was performed to determine the significance of mean values for multiple comparisons at α < 0.05. A principal component analysis was carried out to detail the level of association between the evaluated variables.

## 3. Results

### 3.1. Polyphenol Content and Antioxidant Activity

Table 1 reports the results of the phenolic content and antioxidant activity of EEML, expressed as total phenolic and flavonoid contents (TPC and TFC, respectively), as well as antiradical DPPH^•^ activity and reducing power (RP). The results showed that EEML exhibits high values of TPC and TFC (>200 mg GAE or RE/g, for both). In addition, the results of the antioxidant activity also showed that EEML displays high radical DPPH^•^ inhibition and RP at 100 µg/mL (>80% and >1.0 abs, respectively) in vitro assay. While high antioxidant activity values (*P* < 0.05) were obtained for the used standards, i.e., ascorbic acid (>90% of radical inhibition and >1.0 abs for RP) and BHT (>65% of radical inhibition and >0.7 abs for RP). Furthermore, high correlations between TPC and TFC with respect to the DPPH^•^ and RP assays were observed (TPC vs. TFC, 0.973; TPC vs. DPPH^•^, 0.969; TPC vs. RP, 0.994; TFC vs. DPPH^•^ 0.830; TFC vs. RP, 0.992).

### 3.2. Physicochemical Analysis

Table 2 shows the results of the proximate chemical composition of pork patties. The inclusion of EEML did not modify (*P* > 0.05) the moisture (67.2%), fat (11.1%), ash (1.7%), and protein (22.5%) contents compared to the control group (CN). On the other hand, Table 3 reports the effect of EEML addition in pork patties on physicochemical parameters such as pH and colour (L*, a*, b*, C*, and h*), metmyoglobin formation (MMb), and water holding capacity (WHC). Our results indicated that the treatment × storage time effect was significant for all measurements (*P* < 0.001).

As shown in Table 3, the pH values ranged from 5.85 to 5.92 for all pork patties, which decreased gradually for all treatments until the last day of storage (day 10). At day 10 of storage, all samples treated with the natural antioxidants (EEML1 and EEML2) showed the highest pH values (*P* < 0.05), in comparison with the synthetic antioxidants (Asc and BHT) (pH 5.7) > CN group (pH 5.5). In this study, the results for the colour surface of patties showed that initial L* values (lightness) were not affected by EEML incorporation, and they increased in the CN group during the storage time (*P* > 0.05). At day 10, the samples treated with EEML and synthetic antioxidants presented the lowest L* values (average value 54.1), in comparison with the CN treatment (L* value 56.6) (*P* < 0.05). The initial a* and b* values (redness and yellowness, respectively) showed that EEML incorporation reduced the light pink colour of the samples by 27.8% and increased the b* values by 42.1% in comparison with the CN group (*P* < 0.05). However, these colour parameters decreased and increased, respectively, in the pork patties during storage time (*P* < 0.05). After 10 days of storage, patties from the EEML treatments displayed the highest a* value (>10) and the highest b* value (>20) in comparison with the CN group (*P* < 0.05). In addition, initial C* and h* values were increased (*P* < 0.05) by EEML2 addition (19.1% and 37.8%, respectively). However, C* values decreased in pork patties during storage time (*P* < 0.05); while h* values increased (*P* < 0.05), except for the EEML treatments (*P* > 0.05). At the end of the storage period, the pork patties from the EEML2 treatment showed the highest (*P* < 0.05) C* and h* values (>20 and > 70, respectively).

Moreover, at day 0 of storage non-significant differences (*P* > 0.05) were observed in MMb formation (<2%). However, after 10 days of storage, samples from the EEML group showed the lowest (*P* < 0.05) MMb values (<40% of formation) in comparison with the other treatments (>70% of formation). The addition of EEML reduced the MMb by 55.2% and 62.1% (EEML1 and EEML2, respectively) when compared with CN (*P* < 0.05). Additionally, non-significant differences (*P* > 0.05) were observed in the initial WHC values (>94%), although these values were reduced during storage time for the CN and synthetic antioxidant treatments (*P* < 0.05) treatments. At the end of the storage period, EEML addition to pork patties preserved the WHC (5%) in comparison with the CN (*P* < 0.05). 

### 3.3. Lipid Oxidation

**Figure 1:**
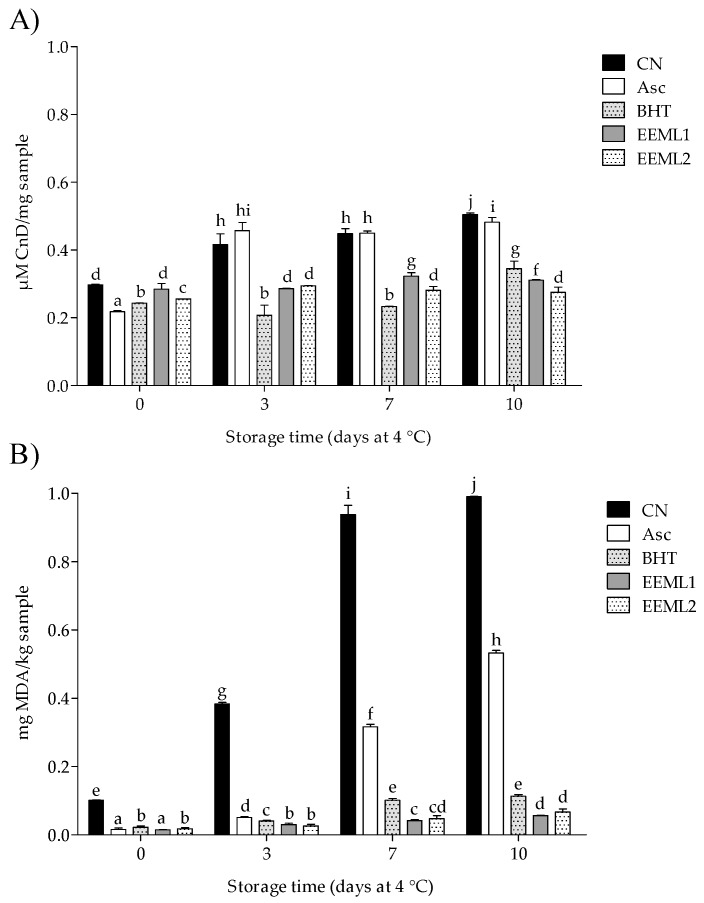
Lipid oxidation levels of pork patties during storage time determined by the conjugated diene formation (CnD) (**A**) and thiobarbituric acid (TBA) (**B**) assays. CN, control; Asc, ascorbic acid; BHT, butylated hydroxytoluene; EEML1, ethanol extract of mesquite leaf at 0.05%; EEML2, ethanol extract of mesquite leaf at 0.1%. Bars with different superscripts (a–j) differ significantly among treatments through the storage time (*P* < 0.05).

The results obtained for conjugated dienes (CnD) and thiobarbituric acid reactive substances (TBARS) indicated that LOX was significantly affected by treatment × storage time (*P* < 0.001). As shown in Figure 1, at the beginning CnD (CN > EEML1 > EEML2 > BHT >Asc) and TBARS values (CN > BHT > EEML2 >Asc > EEML1) were significantly reduced (*P* < 0.05). Although these values increased during the whole period for all treatments (*P* < 0.05), at the end of storage, pork patties treated with EEML1 treatment presented the lowest CnD values (40% of inhibition), while EEML1 = EEML2 showed the lowest TBARS values (90% of inhibition) when compared with the CN group (*P* < 0.05).

### 3.4. Total Antioxidant Activity

**Figure 2:**
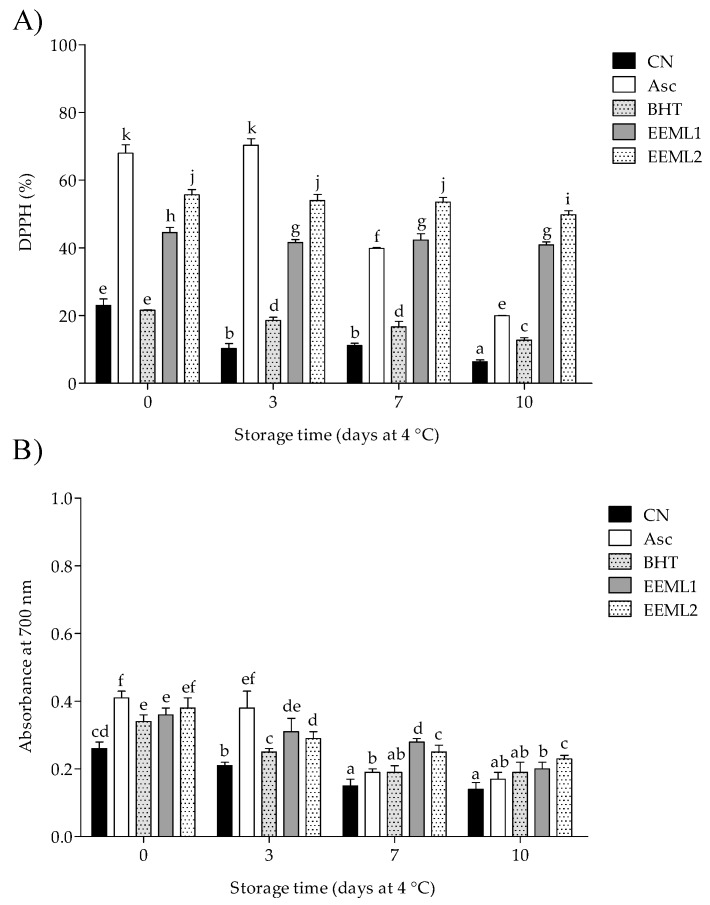
Antioxidant levels of pork patties during storage time determined by the free radical scavenging activity (**A**) and reducing power (**B**) assays. CN, control; Asc, ascorbic acid; BHT, butylated hydroxytoluene; EEML1, ethanol extract of mesquite leaf at 0.05%; EEML2, ethanol extract of mesquite leaf at 0.1%. Bars with different superscripts (a–k) differ significantly between treatments through storage time (*P* < 0.05).

As shown in Figure 2, on the initial day DPPH^•^ inhibition and RP were significantly high (*P* < 0.05) in samples from Asc and EEML treatments (>40% of inhibition, and >35% of reducing activity) in comparison with the CN group. After 10 days of storage, the pork patties treated with ethanol extract of mesquite leaf (EEML1 and EEML2 groups) showed the highest antioxidant activity (>40% of inhibition and >20% of RP) in comparison with the CN treatment (*P* < 0.05).

As shown in Table 4, the results of the sensory evaluation showed that raw pork patties treated with EEML had the lowest scores (EEML2 and EEML1 groups) for colour in comparison with the CN treatment (*P* < 0.05). However, non-significant differences were found in the sensory scores for colour, appearance, odour, flavour, juiciness, fat sensation, and firmness of cooked pork patties among treatments (*P* > 0.05). 

**Figure 3:**
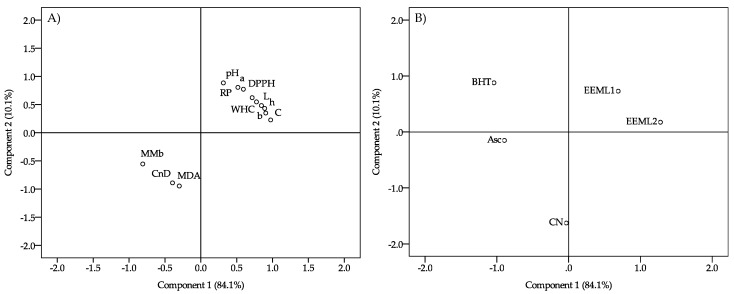
(**A**) Principal component analysis of pork patties and (**B**) loadings graph.

Finally, a principal component analysis was carried out (Figure 3). The first principal component explained 84.1% of the variation, while the second component contributed a further 10.1%; thus, an accumulative 94.2% of the total variation was explained by the first two principal components. The loading plot (Figure 3A) showed that EEML addition increased the pH and a* values, WHC, and antioxidant activity. Furthermore, the MMb, Cnd, and TBARS values were also reduced. In addition, the loading plot (Figure 3B) showed that pork patties from the CN, synthetic antioxidants, and EEML treatments were differentiated. 

## 4. Discussion

The antioxidant activity of extracts obtained from plants is widely known and associated with the polyphenols content, such as hydroxycinnamic acid, anthocyanin, tannin, and flavonoid, which possess the ability to act as a free radical scavenger and ion metal chelator [37]. The measurement of TPC relies on the electron transfer in alkaline medium from the antioxidant (phenolic compound, ArOH) to phosphomolybdic/phosphotungstic acid complexes (colorimetric reagent Folin–Ciocalteu). Whereas the TFC method is based on the formation of a complex between the OH groups from ArOH with aluminium. These assays have been proposed as a standardized method for establishing the quality of natural extracts and measurement of the antioxidant capacity of food products and dietary supplements [25,26]. In agreement with our study, the presence of polyphenols (68–108.3 mg GAE/g) in mesquite leaf extract has been reported [38]. On the other hand, according to Mexican’s regulation, 50 mg GAE/g and 5 mg RE/g are considered the minimum concentration for a natural extract product [39]; which revealed that EEML meets the quality requirements.

Moreover, the antiradical DPPH^•^ activity of ArOH from natural extracts is due to their hydrogen donating ability, and the reaction is based on the reduction of the purple-coloured DPPH^•^ radical to its reduced form 1,1-diphenyl-2-picryl hydrazine, residual pale yellow-coloured: i.e., DPPH^•^ + ArOH → DPPH-H + ArO^•^ [28,40]. On the other hand, in RP assay, the reductants present in the extract promote the reduction of Fe^3+^ to the Fe^2+^ through electron-transfer ability (i.e., Fe^3+^ + ArOH → Fe^2+^ + ArOH^•+^), and high absorbance values (at 700 nm) indicate high RP [41]. In agreement with our study, a strong positive correlation between phenolic content and antioxidant activity has been reported in an extensive range of natural extracts rich in ArOH [42,43]. In this regard, the findings obtained from the present study highlight that EEML is a promising source of ArOH with antioxidant activity, which may be employed as an efficient natural additive for extending the shelf life of fresh meat and meat products.

The effect of EEML on chemical composition and meat quality parameters (pH, colour, MMb, WHC, CnD, and TBARS) during the storage time (4 °C, 10 days, under darkness) was also evaluated. The results obtained indicate that the chemical composition of pork patties treated with EEML and synthetic antioxidant are consistent with data reported by other authors [44,45] concerning pork patties. On the other hand, during the whole period (day 0 to day 10), the pH values of samples ranged from 5.92 to 5.53. The pH is a major parameter related to the quality of fresh meat and meat products. Changes in pH values can affect the chemical (accelerate the myoglobin formation), technological (WHC, cooking weight loss and texture), and sensory properties such as appearance, juiciness, firmness, and colour [46,47].

The colour is a subjective psycho-physical characteristic as it exists only in the observer’s eyes and brain (i.e., it is not a characteristic proper to the object under observation), which is associated with the freshness, flavour, tenderness, safety, storage time, nutritional value, and satisfaction level [48,49,50]. The L* value measures lightness and range from 0 (black) to 100 (white) and is considered the best indicator of PSE (pale, soft, and exudative) and/or DFD (dark, firm, and dry) meat condition. In this regard, L* values above 54 are considered as PSE [51] in pork meat samples. In our study, the initial L* values of pork patties treated with EEML and synthetic antioxidants ranged from 53 to 54. At the end of the storage period, our results showed that the addition of EEML and synthetic antioxidants maintained the lightness 4.4% below in comparison with the CN group. 

The rate of redness (a*) is more useful than that of the yellowness (b*) when the colour surface due to a* values measures colour changes between red and green [50]. At the end of the storage time, our results showed that EEML treatments maintained 25% of the red colour of pork patties in comparison with the CN treatment. In agreement with our results, it has been found that ethanol extract of green tea leaf at 0.1% increased 16.3% of the redness of raw pork patties stored at 4 °C during 10 days of storage [52]. In addition, the Chroma values (C) have been described as a good colour change indicator, and this index decreases when the red index (a*) and pH values decrease, while the hue angle (h*) and MMb increase [44]. The results obtained indicated that the initial C* and h* values were affected by EEML addition, which could be associated to the colouration pigment provided by this natural extract.

Myoglobin (Mb) is the principal protein responsible for the fresh meat colour [47]. Meat discolouration results from oxidation of both ferrous myoglobin derivatives to ferric ion: Oxymyoglobin (OMb) + (oxygen consumption or low O_2_ partial pressure) − e^−^ → Metmyoglobin (MMb) + O_2_^−^ [47]. In our study, prolonged storage of meat under oxygen significantly increased the transformation of OMb (bright pink colour) into MMb (green–brown colour). At the end of the storage time, the lowest MMb values were observed in EEML treatments (<40%), which may be due to the richness in ArOH of EEML. In this regard, it has been reported that a meat product with 40% of MMb formation is rejected by the consumer [53]. On the other hand, the addition of plant extract in the formulation of pork patties could change the colour of the meat samples and increase apparently MMb contents [36]. However, it has been reported that ArOH (kaempferol, myricetin, and quercetin at 300 µmol/L; sinapic acid, catechin, taxifolin, morin, and ferulic acid at 300 µmol/L) exert potential MMb reduction to OMb (bright red protein of meat), suggesting that the flavanol structure had high RP levels [54].

In pork meat, a decrease in pH values promotes myofibrillar protein denaturation, as well as the ability of protein–water linkage and decreased WHC [55]. At the end of the storage time, our results showed that the inclusion of EEML increased the WHC by 5.2% in comparison with the CN group. It has been reported that natural antioxidants increase water retention when acting against free radicals or reactive oxygen species (^•^OH, hydroxyl; O_2_^•−^, superoxide; and H_2_O_2_, hydrogen peroxide), which affect proteins and leads to meat structure modification [38,55]. Thus, plant extracts may protect muscle fibres and decrease moisture loss.

Dietary lipids, naturally occurring in raw meat or added during meat processing, play an important role in food nutrition and flavour [56,57,58,59]. On the other hand, LOX resulted in the formation of conjugated dienes and aldehydes, among others [60,61,62]. At the end of the storage period, our results showed that EEML addition decreased the CnD and TBARS values (40% and 90%, respectively) in comparison with the CN group. In agreement with our results, a reduction in MDA formation (85%) has been reported in raw pork patties (stored at 4 °C for 15 days) treated with avocado extract rich in ArOH [63]. In addition, an MDA reduction (23.2%) was observed in raw pork meat treated with ethanol extract of green tea leaf at 0.1% after ten days of storage [52]. 

The balance between endogenous and exogenous antioxidant and pro-oxidant substances determines the oxidative stability of meat [9]. on the initial day, our results showed that EEML increased the antioxidant status of pork meat (i.e., antiradical and RP); while at the end of the storage time, samples from the EEML treatments showed the highest antioxidant activity (>40% of radical inhibition, and >20% of RP, respectively) compared to the CN group. In agreement with our results, it has been reported that lotus rhizome knot (LRK) and lotus leaf (LL) extract at 3% increased the antioxidant status of raw pork patties (60% of DPPH^•^ inhibition and >80% of RP, by both extracts) stored at 4 °C, in comparison with a control group [36].

Additionally, our results showed that pork patties treated with EEML could be consumed without any problem regarding sensory quality. In agreement with our results, it has been reported that ginger powder at 1% and 2% could be incorporated into pork patties without any effect on their sensorial attributes (appearance, juiciness, flavour, acceptability, or global evaluation, among others), enhance the shelf life and provide a healthy meat product [55].

## 5. Conclusions

EEML incorporation in pork patties resulted in a significant increase in pH values, colour stability, WHC, and antioxidant levels, as well as sensory acceptability. Moreover, EEML improved MMb and LOX stability of treated pork patties during the storage time. We can conclude that EEML can be applied in the meat industry to improve quality and prevent the oxidation process during storage.

## Figures and Tables

**Table 1:** Antioxidant properties of ethanol extract of mesquite leaf (EEML).

**Polyphenol Content**	
TPC (mg GAE/g)	278.5 ± 8.5
TFC (mg RE/g)	226.8 ± 8.3
**Antioxidant Activity**	
**DPPH^•^ (%)**	
100 µg/mL	85.3 ± 0.3 ^b^
50 µg/mL	74.8 ± 4.0 ^a^
25 µg/mL	68.9 ± 6.6 ^a^
Asc (25 µg/mL)	96.3 ± 2.3 ^c^
BHT (50 µg/mL)	69.3 ± 4.1 ^a^
**RP (absorbance at 700 nm)**	
100 µg/mL	1.1 ± 0.2 ^b^
Asc (25 µg/mL)	1.4 ± 0.1 ^b^
BHT (50 µg/mL)	0.7 ± 0.2 ^a^

TPC, total phenolic content; GAE, gallic acid equivalents; TFC, total flavonoids content; RE, rutin equivalents; RP, reducing power; Asc, ascorbic acid; BHT, butylated hydroxytoluene. Values expressed as mean ± standard deviation of at least three independent experiments. Different superscripts (a–c) differ significantly (*P* < 0.05).

**Table 2:** Chemical composition of pork patties.

Treatment	Moisture	Fat	Ash	Protein
CN	66.98 ± 0.58	11.04 ± 0.89	1.67 ± 0.11	20.54 ± 0.74
Asc	67.59 ± 0.86	11.02 ± 0.50	1.68 ± 0.02	22.47 ± 0.54
BHT	67.47 ± 0.70	11.10 ± 0.49	1.66 ± 0.04	23.10 ± 0.45
EEML1	67.06 ± 0.14	11.29 ± 1.08	1.67 ± 0.03	22.96 ± 0.13
EEML2	66.89 ± 1.26	11.05 ± 0.83	1.68 ± 0.06	23.52 ± 0.87
*P*-value	0.765	0.992	0.995	0.246

Values expressed as mean ± standard deviation of at least three independent experiments. CN, control; Asc, ascorbic acid; BHT, butylated hydroxytoluene; EEML1, ethanol extract of mesquite leaf at 0.05%; EEML2, ethanol extract of mesquite leaf at 0.1%.

**Table 3:** Meat quality attributes of pork patties during storage time.

Item	Treat	Storage Time (days)			
		0	3	7	10
pH	CN	5.86 ± 0.02 ^e^	5.78 ± 0.01 ^c^	5.76 ± 0.04 ^bc^	5.53 ± 0.01 ^a^
	Asc	5.85 ± 0.05 ^e^	5.87 ± 0.02 ^e^	5.82 ± 0.01 ^d^	5.72 ± 0.03 ^b^
	BHT	5.87 ± 0.01 ^e^	5.88 ± 0.01 ^e^	5.86 ± 0.04 ^e^	5.76 ± 0.01 ^bc^
	EEML1	5.92 ± 0.01 ^f^	5.88 ± 0.01 ^e^	5.88 ± 0.01 ^e^	5.84 ± 0.02 ^e^
	EEML2	5.91 ± 0.01 ^f^	5.88 ± 0.01 ^e^	5.87 ± 0.02 ^e^	5.85 ± 0.01 ^e^
L*	CN	53.37 ± 0.50 ^a^	53.06 ± 0.35 ^a^	53.22 ± 0.41 ^a^	56.60 ± 0.77 ^b^
	Asc	53.82 ± 0.88 ^a^	54.68 ± 0.56 ^a^	54.19 ± 0.35 ^a^	54.25 ± 1.22 ^a^
	BHT	54.63 ± 0.79 ^a^	54.39 ± 1.02 ^a^	54.35 ± 0.29 ^a^	53.75 ± 0.50 ^a^
	EEML1	54.52 ± 0.48 ^a^	54.31 ± 0.75 ^a^	53.99 ± 0.65 ^a^	54.40 ± 0.56 ^a^
	EEML2	54.39 ± 0.58 ^a^	53.73 ± 0.41 ^a^	53.92 ± 0.22 ^a^	54.03 ± 0.80 ^a^
a*	CN	15.78 ± 1.08 ^d^	15.74 ± 1.22 ^d^	14.03 ± 0.46 ^cd^	7.23 ± 0.40 ^a^
	Asc	15.66 ± 0.44 ^d^	14.14 ± 0.25 ^cd^	14.88 ± 0.51 ^cd^	9.99 ± 0.71 ^b^
	BHT	14.82 ± 1.06 ^cd^	14.73 ± 0.76 ^cd^	13.78 ± 0.96 ^c^	9.05 ± 0.79 ^b^
	EEML1	12.61 ± 1.25 ^c^	12.59 ± 1.14 ^c^	12.79 ± 1.13 ^c^	10.09 ± 0.76 ^b^
	EEML2	10.19 ± 1.41 ^bc^	10.56 ± 0.71 ^bc^	10.31 ± 0.37 ^b^	9.18 ± 0.41 ^b^
b*	CN	15.01 ± 0.99 ^a^	16.02 ± 1.10 ^a^	16.77 ± 0.28 ^a^	17.37 ± 0.15 ^b^
	Asc	15.18 ± 0.58 ^a^	15.02 ± 1.08 ^a^	15.54 ± 0.32 ^a^	14.52 ± 0.68 ^a^
	BHT	15.32 ± 1.05 ^a^	15.90 ± 0.90 ^a^	15.96 ± 0.78 ^a^	14.97 ± 0.92 ^a^
	EEML1	20.75 ± 1.02 ^c^	21.45 ± 1.10 ^c^	21.56 ± 0.84 ^c^	20.14 ± 0.66 ^c^
	EEML2	25.94 ± 0.10 ^e^	25.74 ± 0.44 ^e^	24.40 ± 0.93 ^de^	22.67 ± 1.11 ^cd^
C*	CN	21.47 ± 1.13 ^bc^	19.83 ± 1.69 ^b^	19.73 ± 0.89 ^b^	15.37 ± 0.47 ^a^
	Asc	21.71 ± 0.61 ^b^	20.83 ± 2.03 ^b^	21.87 ± 0.39 ^b^	16.87 ± 0.89 ^a^
	BHT	20.66 ± 1.57 ^b^	21.92 ± 1.02 ^bc^	20.43 ± 2.02 ^b^	15.73 ± 1.16 ^a^
	EEML1	23.61 ± 1.61 ^bc^	23.45 ± 1.07 ^bc^	23.96 ± 0.58 ^c^	21.77 ± 1.74 ^b^
	EEML2	26.53 ± 1.67 ^d^	26.97 ± 0.60 ^d^	26.06 ± 0.94 ^d^	23.61 ± 1.06 ^bc^
H*	CN	43.57 ± 0.95 ^a^	44.52 ± 1.24 ^a^	46.47 ± 1.34 ^a^	55.69 ± 1.09 ^b^
	Asc	43.82 ± 0.53 ^a^	42.53 ± 1.70 ^a^	45.50 ± 0.38 ^a^	53.25 ± 1.10 ^b^
	BHT	44.50 ± 1.15 ^a^	45.83 ± 1.06 ^a^	46.40 ± 1.30 ^a^	56.15 ± 1.60 ^b^
	EEML1	61.23 ± 2.17 ^c^	60.19 ± 1.20 ^c^	60.4+ ± 2.07 ^c^	54.56 ± 1.19 ^b^
	EEML2	70.05 ± 1.49 ^d^	70.50 ± 1.46 ^d^	69.59 ± 1.90 ^d^	72.93 ± 1.64 ^d^
MMb	CN	1.00 ± 0.18 ^a^	7.90 ± 0.45 ^b^	71.22 ± 3.10 ^h^	91.78 ± 2.93 ^k^
	Asc	1.70 ± 0.70 ^a^	14.67 ± 1.30 ^c^	71.70 ± 1.38 ^h^	70.12 ± 2.43 ^h^
	BHT	1.40 ± 0.27 ^a^	8.51 ± 1.98 ^b^	79.83 ± 2.11 ^i^	80.97 ± 2.19 ^j^
	EEML1	1.40 ± 0.18 ^a^	6.03 ± 0.60 ^b^	28.75 ± 2.65 ^e^	41.15 ± 2.63 ^g^
	EEML2	1.20 ± 0.27 ^a^	7.73 ± 1.46 ^b^	17.19 ± 1.26 ^d^	34.81 ± 2.68 ^f^
WHC	CN	95.83 ± 0.85 ^b^	95.08 ± 0.62 ^b^	95.77 ± 1.00 ^b^	91.20 ± 0.88 ^a^
	Asc	94.26 ± 0.90 ^b^	94.80 ± 0.91 ^b^	94.61 ± 1.05 ^b^	92.20 ± 0.64 ^a^
	BHT	94.92 ± 1.34 ^b^	95.57 ± 1.01 ^b^	94.98 ± 0.22 ^b^	95.42 ± 0.59 ^b^
	EEML1	95.04 ± 0.80 ^b^	95.34 ± 1.65 ^b^	95.68 ± 1.36 ^b^	95.86 ± 0.47 ^b^
	EEML2	94.61 ± 0.98 ^b^	95.25 ± 0.32 ^b^	95.75 ± 0.27 ^b^	95.96 ± 0.26 ^b^

Values expressed as mean ± standard deviation of at least three independent experiments. L*, lightness, a*, redness, b*, yellowness, C*, Chroma, h*, hue angle. CN, control; Asc, ascorbic acid; BHT, butylated hydroxytoluene; EEML1, ethanol extract of mesquite leaf at 0.05%; EEML2, ethanol extract of mesquite leaf at 0.1%. Different superscripts (a–k) differ significantly between treatments through storage time (*P* < 0.05).

**Table 4:** Sensory evaluation scores of pork patties.

Item	Treatments			*P*-Value
	CN	EEML1	EEML2	
**Raw patties**				
Colour	8.8 ± 1.4 ^c^	5.5 ± 1.2 ^b^	4.3 ± 1.3 ^a^	0.013
Appearance	6.1 ± 1.0	5.6 ± 1.0	5.4 ± 0.4	0.114
**Cooked patties**				
Colour	5.4 ± 1.0	5.1 ± 1.1	4.7 ± 1.4	0.775
Appearance	5.7 ± 1.1	5.4 ± 1.0	4.8 ± 1.4	0.656
Odour	5.4 ± 1.3	4.7 ± 1.2	5.6 ± 1.2	0.663
Flavour	5.5 ± 1.2	5.4 ± 1.2	4.7 ± 1.2	0.689
Juiciness	4.8 ± 1.2	5.6 ± 0.7	4.7 ± 1.3	0.576
Fat sensation	5.5 ± 1.5	5.5 ± 1.0	4.7 ± 1.4	0.706
Firmness	5.3 ± 1.4	5.7 ± 1.2	5.1 ± 1.3	0.852

Values expressed as mean ± standard deviation of at least three independent experiments. CN, control; EEML1, mesquite leaf extract at 0.05%; EEML2, mesquite leaf extract at 0.1%. Different superscripts (a–c) differ significantly between treatments within the same sensory attribute (*P* < 0.05).
